# Hybrid Evidence-Informed Synthesis of Resting-State Functional Connectivity Alterations in Mild Traumatic Brain Injury

**DOI:** 10.3390/brainsci16060557

**Published:** 2026-05-23

**Authors:** Ioannis Mavroudis, Foivos Petridis, Alin Ciobica, Roxana O. Cojocariu, Dimitrios Kazis, Ahmed Adel Mansour Kamar, Cătălina Ionescu, Diana Gheban, Catalin Morosan, Bogdan Gurzu, Otilia Novac, Bogdan Novac

**Affiliations:** 1 Neuroscience Department, Leeds Teaching Hospitals NHS Trust, Leeds LS1 3EX, UK; i.mavroudis@nhs.net; 2 Romanian Academy of Scientists (Academia Oamenilor de Știință din România), 050044 Bucharest, Romania; 3 Third Department of Neurology, Aristotle University of Thessaloniki, GR-54124 Thessaloniki, Greece; foivos.petridis@gmail.com (F.P.); dimitrios.kazis@gmail.com (D.K.); 4 Department of Biology, Faculty of Biology, “Alexandru Ioan Cuza” University of Iasi, 700505 Iasi, Romania; alin.ciobica@uaic.ro; 5 “Ioan Hăulică” Institute, “Apollonia” University of Iași, 700511 Iasi, Romania; catalinaionescu81@yahoo.com (C.I.); diana.gheban@yahoo.com (D.G.); 6 “Olga Necrasov” Center, Biomedical Research Group, Romanian Academy, 700481 Iasi, Romania; 7 CENEMED Platform for Interdisciplinary Research, Grigore T. Popa University of Medicine and Pharmacy, 700115 Iasi, Romania; 8 Department of Biological and Morphological Sciences, Faculty of Medicine and Biological Science, Stefan Cel Mare University of Suceava, 720229 Suceava, Romania; 9 Medical Department, Gulf of Suez Petroleum Company (GUPCO)—Cairo Office, Cairo 11511, Egypt; 10 Department of Orthopedics and Traumatology, Clinical Recovery Hospital (Recuperare), 700661 Iasi, Romania; 11 Doctoral School, Faculty of Biology, “Alexandru Ioan Cuza” University of Iasi, 700505 Iasi, Romania; 12 Faculty of Medicine, Grigore T. Popa University of Medicine and Pharmacy, 700115 Iasi, Romania; morosangeorgecatalin@yahoo.com (C.M.); bgurzu@yahoo.com (B.G.);

**Keywords:** mild traumatic brain injury, resting-state fMRI, functional connectivity, default mode network, post-concussion syndrome, large-scale brain networks, neuroimaging synthesis

## Abstract

**Highlights:**

**What are the main findings?**

- Convergent evidence indicates preferential disruption of the default mode network, executive frontal circuits, and hippocampal regions following mild traumatic brain injury.
- Hypoconnectivity is more frequently reported than hyperconnectivity, though directional variability appears influenced by injury stage and neurovascular factors.

**What are the implications of the main findings?**

- Mild traumatic brain injury is best conceptualized as a disorder of distributed network dysfunction rather than focal structural damage.
- Standardized, multimodal, and longitudinal imaging approaches are required before resting-state connectivity measures can achieve clinical translation.

**Abstract:**

Background: Mild traumatic brain injury (mTBI) is frequently followed by persistent cognitive, affective, and sensory complaints despite unremarkable conventional structural imaging. Resting-state functional MRI (rs-fMRI) has been increasingly employed to detect subtle alterations in large-scale brain networks. However, variability in analytical approaches and the potential influence of neurovascular factors complicate interpretation of BOLD-derived connectivity findings. Objective: This study provides a focused, evidence-informed synthesis integrating umbrella review principles with a targeted narrative analysis of recent high-quality rs-fMRI studies in mild traumatic brain injury (mTBI). Rather than a comprehensive systematic review, the aim was to identify convergent patterns of network dysfunction while critically examining methodological constraints, including neurovascular confounds and variability in analytical approaches. Conclusions: This synthesis supports a network-level model of mTBI characterized by distributed connectivity disturbances. However, given the limited number of eligible studies and substantial methodological heterogeneity, findings should be interpreted as qualitative convergence rather than quantitative generalization. Future longitudinal, multimodal, and standardized imaging approaches are required to clarify the translational relevance of rs-fMRI findings.

## 1. Introduction

Mild traumatic brain injury (mTBI), often referred to as concussion, represents the most common form of traumatic brain injury worldwide and constitutes a substantial public health burden [1,2]. Although the majority of individuals recover within days to weeks, a clinically meaningful subset experiences persistent symptoms that extend beyond the expected recovery window. These symptoms—frequently grouped under the term post-concussion syndrome (PCS)—may include attentional difficulties, slowed information processing, memory complaints, emotional lability, and sensory hypersensitivity.

A central clinical challenge lies in the fact that such symptoms often arise despite normal findings on conventional structural imaging. Standard CT and routine MRI sequences are typically insensitive to diffuse or network-level disturbances, creating a mismatch between subjective symptom burden and objective radiological findings. This discrepancy has motivated increasing interest in advanced neuroimaging approaches capable of detecting subtle alterations in brain organization that are not visible using traditional structural techniques.

Resting-state functional magnetic resonance imaging (rs-fMRI) has become a widely used method for investigating large-scale brain organization in both health and disease.

Rather than probing task-evoked activation, rs-fMRI examines spontaneous low-frequency fluctuations in the blood-oxygen-level-dependent (BOLD) signal that are temporally correlated across distributed cortical and subcortical regions. These intrinsic correlations give rise to reproducible functional networks, including the default mode network (DMN), salience network, and frontoparietal control network [3,4].

Among these systems, the DMN has received particular attention in the context of mTBI. Core DMN nodes—such as the posterior cingulate cortex and precuneus—play central roles in internally directed cognition, attentional regulation, and episodic memory. Disruption of these functions is frequently reported following concussion, making the DMN a plausible candidate for network-level vulnerability [5,6].

Although rs-fMRI has expanded our understanding of functional alterations following mTBI, the literature remains characterized by considerable variability. Differences in injury mechanism, time since trauma, symptom burden, and participant demographics intersect with methodological diversity in acquisition parameters, preprocessing strategies, motion correction procedures, and statistical thresholding. These sources of heterogeneity complicate cross-study comparison and contribute to apparently divergent findings across cohorts.

A systematic review by Eierud et al. [5] underscored this issue, noting that while alterations in default mode and frontoparietal networks were frequently reported, the direction and spatial extent of connectivity changes varied substantially. Similarly, longitudinal investigations have suggested that network alterations may evolve over time, with early hyperconnectivity in some regions transitioning to reduced connectivity in later phases [7]. Together, these observations suggest that mTBI-related dysfunction may be temporally dynamic rather than static, and that apparent inconsistencies may partly reflect differences in injury stage and analytic approach rather than true contradiction.

Evidence from diffusion tensor imaging (DTI) provides complementary context for interpreting functional connectivity findings. In a meta-analysis of DTI studies, Aoki et al. [8] reported consistent reductions in fractional anisotropy within the corpus callosum and other major white matter tracts in individuals with mTBI, suggesting the microstructural disruption of interhemispheric and frontoparietal pathways. Such structural alterations offer a plausible substrate for the functional dysconnectivity observed in rs-fMRI investigations.

While structural and functional measures capture distinct aspects of brain organization, converging abnormalities across modalities support the view that mTBI affects distributed networks rather than isolated cortical regions. This network-level perspective provides an important conceptual framework for interpreting rs-fMRI findings within a broader neurobiological context.

Within the rs-fMRI literature, alterations in connectivity involving core default mode network hubs—particularly the posterior cingulate cortex and precuneus—have been among the most frequently reported findings in mTBI cohorts [5,7]. These regions function as integrative nodes linking memory, attention, and self-referential processing, domains commonly affected in post-concussion syndrome.

Beyond the DMN, studies have also described disruptions in frontoparietal control circuits, limbic structures such as the hippocampus, and nodes of the salience network, including the insula. However, the reported directionality of connectivity changes has varied, with some investigations describing reduced coherence and others reporting transient increases, potentially reflecting compensatory recruitment or stage-dependent reorganization. Such variability underscores the importance of contextualizing findings within injury phase, symptom burden, and analytic strategy.

Notably, not all rs-fMRI investigations have demonstrated significant group-level differences following mTBI. Null or mixed findings are frequently attributed to variability in sample composition, injury timing, symptom severity, and statistical thresholding. These discrepancies highlight both the sensitivity and the methodological fragility of resting-state connectivity analyses, reinforcing the need for structured evidence synthesis that can identify patterns of convergence across heterogeneous designs.

In parallel, analytic strategies have evolved beyond traditional seed-based connectivity approaches to include graph theoretical modeling and measures of local signal synchrony. These methods aim to characterize not only pairwise correlations but also global network topology and efficiency. Although such approaches have provided additional insights into network organization after mTBI, they further increase methodological diversity across studies, complicating synthesis and comparison.

Given the methodological heterogeneity and the expanding body of the literature, a structured synthesis that integrates high-level review evidence with recent well-characterized original studies is warranted. The present work adopts a hybrid approach: first, it presents an umbrella synthesis of systematic reviews and meta-analyses, addressing functional connectivity alterations in mTBI; second, it presents the focused narrative integration of recent rs-fMRI investigations selected for methodological rigor and clinical relevance.

The objectives are threefold: (1) to identify convergent patterns of network-level dysfunction across evidence tiers; (2) to examine their relationship to core domains of post-concussion symptomatology; and (3) to critically evaluate methodological constraints, including potential neurovascular confounds and the relative contribution of resting-state versus task-based paradigms. Through this approach, we aim to clarify the extent to which network-level alterations in mTBI demonstrate reproducible convergence despite analytic and clinical heterogeneity.

## 2. Materials and Methods

This review followed the general reporting principles of PRISMA 2020 for evidence synthesis. The design combined two complementary components: (1) an umbrella-level synthesis of published systematic reviews and meta-analyses addressing functional neuroimaging in mild traumatic brain injury (mTBI), and (2) a targeted narrative synthesis of recent original resting-state fMRI (rs-fMRI) investigations not captured within earlier meta-analytic work.

Electronic searches were conducted in PubMed/MEDLINE, Embase, and Scopus from January 2010 through April 2025. The start date was selected to reflect the widespread adoption of resting-state network methodologies in clinical neuroimaging research. Search terms combined controlled vocabulary and free-text keywords related to mild traumatic brain injury (“mild TBI,” “concussion,” “head injury”) and functional connectivity (“resting-state fMRI,” “rs-fMRI,” “functional connectivity,” “default mode network”), alongside terms identifying review-level evidence (“systematic review,” “meta-analysis”).

For the umbrella component, inclusion criteria were: (1) peer-reviewed systematic reviews or meta-analyses examining functional neuroimaging findings in mTBI or repetitive head impact populations; (2) explicit reporting of resting-state connectivity findings; and (3) inclusion of adolescent or adult samples. Reviews restricted exclusively to structural imaging without functional analysis, or focusing primarily on moderate or severe TBI, were excluded.

For the narrative component, original rs-fMRI studies published from 2020 onward were screened for methodological rigor, including clearly described preprocessing pipelines, motion correction procedures, and appropriate statistical correction for multiple comparisons. These studies were selected to contextualize and extend umbrella-level findings rather than to provide quantitative aggregation.

Methodological constraints, including neurovascular confounds, motion-related artifacts, preprocessing variability, and injury-stage heterogeneity, were systematically identified during data extraction and grouped into predefined categories. These factors were not quantitatively controlled but were incorporated into a structured narrative synthesis framework. A vote-counting approach was adopted due to substantial heterogeneity in study design, analytic methods, and reporting metrics, which precluded meaningful statistical pooling.

### 2.1. Data Extraction and Evidence Integration

Two reviewers independently screened titles and abstracts for eligibility, followed by full-text evaluation. Disagreements were resolved through discussion and consensus. From each eligible review, we extracted study characteristics (publication year, sample composition, imaging modality), analytic approach (e.g., seed-based connectivity, independent component analysis, graph metrics), and principal findings regarding network-level alterations.

Rather than performing pooled quantitative meta-analysis, findings were integrated descriptively to identify recurring patterns of network involvement and directionality of connectivity changes. For the narrative component, recent original studies were examined, with attention paid to sample size, injury timing, analytic transparency, and reported associations between connectivity measures and clinical outcomes.

The present study was designed as a focused, evidence-informed synthesis rather than a comprehensive systematic review. Inclusion criteria were intentionally restrictive in order to prioritize methodological transparency, analytical rigor, and relevance to resting-state functional connectivity. Consequently, the final number of included studies reflects selective curation of high-quality evidence rather than exhaustive coverage of the literature.

In addition, methodological constraints and potential confounding factors were systematically identified during data extraction. These included neurovascular influences on BOLD signal interpretation, motion-related artifacts, preprocessing variability (e.g., global signal regression, filtering strategies), statistical thresholding, and injury-stage heterogeneity. These factors were not quantitatively controlled but were categorized into predefined domains and incorporated into a structured narrative synthesis framework.

Given the substantial heterogeneity across studies in terms of imaging acquisition parameters, analytic methodologies, outcome reporting, and absence of standardized effect-size metrics, formal quantitative pooling was not feasible. Therefore, a vote-counting approach was adopted as a descriptive method to identify patterns of convergence in reported connectivity alterations across studies. This approach reflects the frequency of reported findings rather than their magnitude or statistical strength. Accordingly, the present work should be interpreted as a structured qualitative synthesis rather than a formal quantitative meta-analysis.

The relative contributions of resting-state versus task-based paradigms were evaluated through comparative interpretation of findings reported in the included literature, rather than formal quantitative comparison.

### 2.2. Spatial Visualization of Reported Regions

For descriptive visualization, Montreal Neurological Institute (MNI) coordinates reported in the primary literature were extracted where available. When peak coordinates were not explicitly provided, approximate anatomical centroids were derived from reported regions.

These locations were visualized using the Nilearn Python library (Version 0.13.1) on a standard MNI152 template to illustrate spatial convergence across studies. This procedure was intended solely for graphical representation and does not constitute coordinate-based meta-analysis.

### 2.3. Quality Assessment

The methodological quality of included systematic reviews and meta-analyses was assessed using AMSTAR-2, a validated instrument for evaluating review-level evidence in healthcare research. Domains assessed included comprehensiveness of search strategy, protocol registration, risk-of-bias evaluation, and transparency of synthesis methods.

For original rs-fMRI studies included in the narrative component, quality appraisal focused on sample characterization, clarity of preprocessing steps, motion correction procedures, and application of multiple-comparison correction. Given the heterogeneity of analytic approaches in functional neuroimaging, a formal pooled quality score was not calculated; instead, methodological strengths and limitations were described narratively.

## 3. Results

### 3.1. Study Selection

The structured search process identified seven studies meeting eligibility criteria. Four were systematic reviews or meta-analyses forming the umbrella component, and three were original resting-state fMRI investigations incorporated into the narrative synthesis.

**Figure 1:**
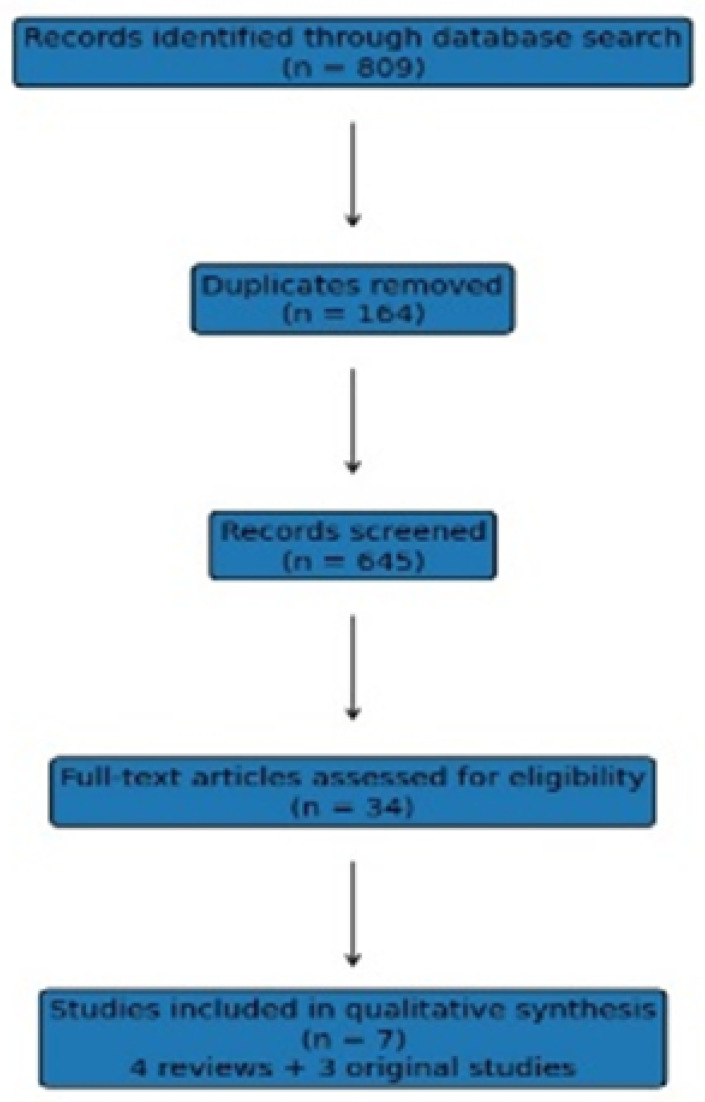
PRISMA flow diagram of study selection.

The study selection process is summarized in Figure 1.

This diagram illustrates the identification, screening, and inclusion process of studies in accordance with PRISMA 2020 guidelines. A total of 809 records were identified through database searches, and seven studies met eligibility criteria. These comprised four systematic reviews/meta-analyses, forming the umbrella component, and three original rs-fMRI investigations, included in the narrative synthesis.

The characteristics of the included studies are presented in Table 1 (umbrella component) and Table 2 (original rs-fMRI studies).

This table summarizes review-level evidence addressing functional connectivity alterations in mTBI.

The umbrella-level studies provided broad characterization of neuroimaging findings in mTBI, whereas the narrative component allowed closer examination of recent cohort-level rs-fMRI results not fully captured in earlier reviews.

**Table 2:** Original resting-state fMRI studies included in narrative synthesis.

Study	Year	Sample	Methods/Metrics	Key Findings	Clinical Relevance
Shi et al. [11]	2021	mTBI patients vs. controls	Regional homogeneity (ReHo); graph theoretical metrics	Reduced ReHo in temporal regions and cerebellum; decreased global efficiency in frontal networks	Executive dysfunction associated with altered frontal network topology
Kagialis et al. [12]	2024	Chronic mTBI vs. controls	Seed-based connectivity; hemodynamic–functional coupling analysis	Bilateral hippocampal hypoconnectivity; increased parietal connectivity; functional–hemodynamic uncoupling	Connectivity alterations associated with neurocognitive and mental health indices
Dogra et al. [10]	2024	mTBI vs. controls	Resting-state functional connectivity analysis with multiple-comparison correction	No statistically significant connectivity differences after correction	Highlights influence of analytic thresholds and statistical power
Vedaei et al. [13]	2022	60 chronic mTBI; 40 controls	fALFF, ReHo, degree centrality (DC), VMHC, FCS; machine learning classification	Multimetric features distinguished mTBI from controls with high discriminative performance	Suggests potential biomarker utility using multimodal rs-fMRI metrics

This table presents cohort-level rs-fMRI investigations, incorporated into the focused narrative component. References correspond to studies.

### 3.2. Methodological Quality of Included Reviews

The methodological quality of the four systematic reviews was evaluated using AMSTAR-2 criteria. Overall, most reviews fulfilled core domains related to comprehensive search strategies and duplicate study selection. However, protocol preregistration and formal publication bias assessment were inconsistently reported.

**Figure 2:**
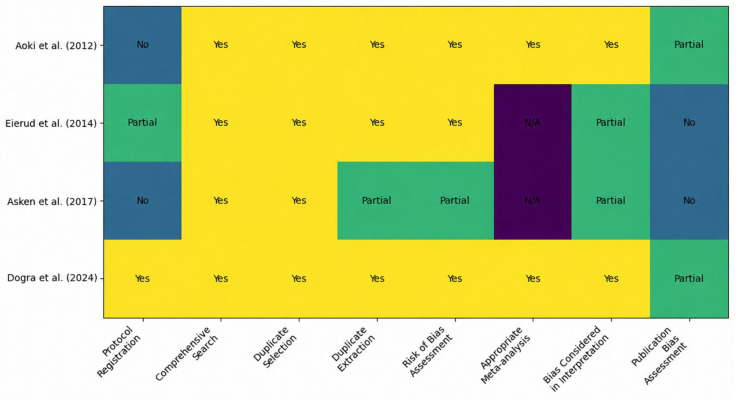
Methodological quality assessment of included systematic reviews (AMSTAR-2 domains) [5,8,9,10].

A domain-level overview of the quality assessment is shown in Figure 2.

This heatmap presents domain-specific methodological appraisal of the included systematic reviews using AMSTAR-2 criteria. Each cell indicates whether a given methodological domain was fulfilled (“Yes”), partially fulfilled (“Partial”), not fulfilled (“No”), or not applicable (“N/A”). No overall numerical score was calculated, in accordance with the recommendations of AMSTAR-2.

Importantly, no aggregate quality score was calculated, in accordance with the recommendations of AMSTAR-2. Instead, individual domains were evaluated to provide a transparent appraisal of methodological strengths and limitations.

### 3.3. Direction and Frequency of Connectivity Changes

Across included studies, reduced functional connectivity was more frequently reported than increased connectivity. Hypoconnectivity was most consistently observed within the default mode network (DMN), particularly in posterior cingulate and precuneus regions. Frontal executive regions (DLPFC) and limbic structures, including the hippocampus, were also recurrently implicated.

**Figure 3:**
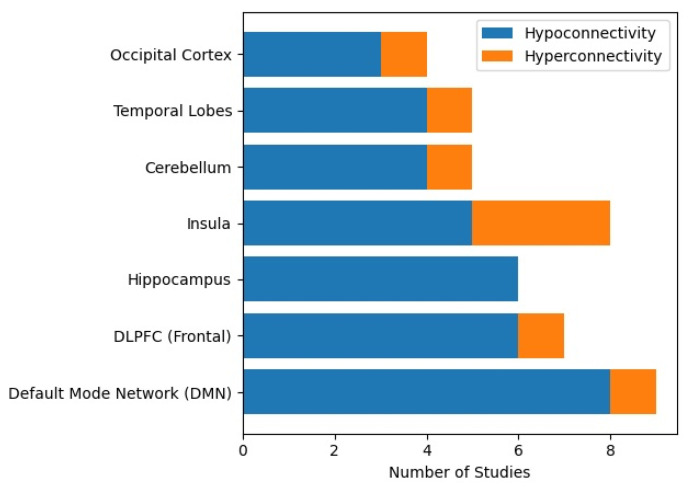
Network-level functional connectivity alterations in mild traumatic brain injury.

A consolidated overview of network-level alterations, integrating both directionality and total frequency, is presented in Figure 3.

Stacked horizontal bars represent the number of included studies reporting hypoconnectivity and hyperconnectivity within major brain networks. Total bar length reflects the overall frequency of reported disruption across studies. The default mode network demonstrates the highest convergence of alterations, followed by executive and limbic circuits.

The DMN demonstrated the highest overall number of reported disruptions, followed by executive frontal circuits and hippocampal regions. Reports of hyperconnectivity were less frequent and more variable across studies, often interpreted as phase-dependent or compensatory responses.

Primary sensory networks were comparatively less represented, though occipital and temporal alterations were observed in specific cohorts.

### 3.4. Narrative Synthesis of Recent rs-fMRI Studies

The three original rs-fMRI investigations provided more detailed cohort-level insights (Table 2).

Shi et al. [11] reported reduced regional homogeneity in temporal regions and the cerebellum, alongside decreased global efficiency in frontal networks associated with executive dysfunction.

Kagialis et al. [12] identified bilateral hippocampal hypoconnectivity and increased parietal connectivity, with evidence of functional–hemodynamic uncoupling. In contrast, Dogra et al. [10] found no statistically significant connectivity differences after correction for multiple comparisons, highlighting the influence of analytic thresholds and statistical power on rs-fMRI findings.

Vedaei et al. [13] analyzed multilevel resting-state fMRI metrics in 60 chronic mTBI patients and 40 matched controls. The study demonstrated that combined measures of fractional amplitude of low-frequency fluctuations (fALFF), regional homogeneity (ReHo), degree centrality (DC), voxel-mirrored homotopic connectivity (VMHC), and functional connectivity strength (FCS) can distinguish mTBI from controls using machine learning classifiers, with several features showing high discriminative performance.

These heterogeneous results reinforce both the sensitivity and methodological fragility of resting-state connectivity analyses in mTBI.

### 3.5. Symptom–Network Contextualization

**Figure 4:**
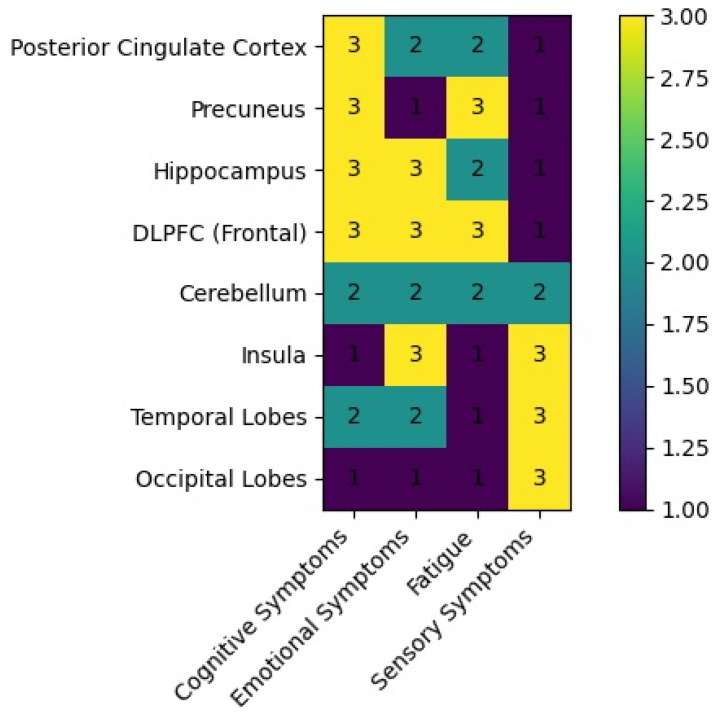
Qualitative symptom–network associations in mild traumatic brain injury.

To facilitate clinical interpretation, frequently implicated regions were descriptively mapped onto core domains of post-concussion symptomatology. The resulting qualitative associations are illustrated in Figure 4.

This heatmap illustrates qualitative associations between frequently implicated brain regions and major post-concussion symptom domains. Values represent the descriptive strength of reported associations across included studies (1 = weak/infrequent, 2 = moderate, 3 = strong/recurrent). These mappings are exploratory and do not reflect quantitative effect-size modeling.

DMN hubs and frontal executive regions demonstrated the most frequently reported qualitative associations with cognitive symptom clusters. Hippocampal and insular alterations were more frequently associated with affective symptoms and fatigue. Occipital and temporal involvement demonstrated greater alignment with sensory disturbances.

These mappings are descriptive and do not represent quantitative effect-size modeling; rather, they reflect convergence across reported symptom associations in the included literature.

## 4. Discussion

### 4.1. Principal Findings

The present hybrid synthesis suggests convergent evidence for distributed large-scale network dysfunction following mild traumatic brain injury (mTBI). Across systematic reviews and original rs-fMRI investigations, alterations were most consistently reported within the default mode network (DMN), executive frontal circuits, and limbic structures, particularly the hippocampus. These findings align with the prior neuroimaging literature, suggesting that mTBI preferentially affects integrative association networks rather than producing focal structural lesions detectable using conventional imaging [5,14].

The predominance of network-level dysfunction supports the growing conceptualization of mTBI as a disorder of disrupted functional integration rather than localized tissue loss [15]. While conventional CT and routine MRI often fail to detect abnormalities in mild cases, functional connectivity analyses reveal subtle but reproducible alterations in large-scale neural systems [7].

### 4.2. Network Vulnerability and Hub Disruption

The recurrent involvement of the DMN is neurobiologically plausible. The DMN comprises metabolically active, highly interconnected cortical hubs, including the posterior cingulate cortex and precuneus, which serve integrative roles in self-referential processing, episodic memory, and attentional modulation [3,4]. Highly connected hubs are thought to be particularly vulnerable to diffuse mechanical forces due to their extensive white matter connectivity and central role in network communication [15,16].

Sharp and colleagues proposed that traumatic brain injury disrupts structural connectivity within hub regions, resulting in widespread secondary functional alterations [15]. Similarly, Buckner et al. described the DMN as a central integrative network characterized by high metabolic demand and extensive long-range projections [3]. This structural–functional vulnerability framework may help to explain the recurrent DMN alterations observed across mTBI cohorts.

The additional involvement of dorsolateral prefrontal cortex (DLPFC) and hippocampal circuits is consistent with common post-concussive cognitive complaints, including deficits in working memory, attentional control, and episodic encoding [17].

### 4.3. Directional Heterogeneity: Hypoconnectivity and Hyperconnectivity

Across included studies, hypoconnectivity was more frequently reported than hyperconnectivity. Reduced functional connectivity within associative networks may reflect impaired network integration or reduced efficiency of long-range communication. However, hyperconnectivity has also been observed, particularly during acute and early subacute phases [18].

This directional variability may reflect dynamic network reorganization over time. Early increases in connectivity have been interpreted as compensatory recruitment or maladaptive over-engagement of residual networks [19]. Longitudinal imaging studies suggest that initial hyperconnectivity may transition to reduced connectivity during chronic stages [18].

Importantly, BOLD-based functional connectivity is influenced not only by neuronal synchrony but also by neurovascular coupling. Alterations in cerebrovascular reactivity following mTBI may influence connectivity metrics independently of synaptic activity [20]. Recent evidence demonstrating functional–hemodynamic uncoupling in chronic mTBI supports the need for cautious interpretation of rs-fMRI findings [12].

Thus, directional heterogeneity likely reflects a combination of injury stage, vascular dynamics, and analytic methodology rather than mutually exclusive biological processes.

### 4.4. Methodological Considerations

Resting-state fMRI findings in mTBI are highly sensitive to preprocessing decisions, including motion correction thresholds, temporal filtering, global signal regression, and network parcellation strategies [21]. Even subtle differences in head motion can spuriously influence functional connectivity estimates [22]. Given that mTBI populations may exhibit increased motion due to symptom burden, rigorous correction strategies are essential.

The variability identified in the AMSTAR-2 appraisal further underscores methodological heterogeneity at the review level. While most systematic reviews demonstrated adequate search strategies, inconsistencies were observed in protocol registration and publication bias assessment. Such variability may contribute to divergent conclusions across reviews.

Additionally, the majority of included studies were cross-sectional. Longitudinal investigations examining network evolution from acute to chronic phases remain limited, constraining causal inference regarding network reorganization trajectories [18].

An important consideration is the method-dependent variability of rs-fMRI findings. Connectivity results are highly sensitive to preprocessing choices, including motion correction thresholds, global signal regression, temporal filtering, and statistical correction strategies. As illustrated by Dogra et al. (2024) [10], findings may lose statistical significance depending on multiple-comparison correction, underscoring the fragility of rs-fMRI-derived metrics as stable biomarkers.

### 4.5. Clinical Interpretation and Translational Limits

The qualitative mapping of network alterations onto post-concussion symptom domains provides a structured interpretative framework, linking distributed network dysfunction to heterogeneous clinical presentation. Disruption within the DMN and DLPFC plausibly contributes to cognitive slowing and attentional deficits, while limbic alterations may relate to affective symptoms and fatigue [19,22].

However, these associations remain correlational. The present synthesis is based on aggregated study-level data and does not permit patient-level diagnostic or prognostic inference. Although rs-fMRI demonstrates sensitivity to subtle network alterations, standardized acquisition protocols and prospective validation studies are required before translation into routine clinical practice [23].

Despite growing interest in network-level biomarkers, several barriers limit clinical implementation. These include the lack of standardized acquisition and preprocessing pipelines, high inter-individual variability, sensitivity to motion and physiological confounds, and limited cross-site reproducibility. Furthermore, the absence of validated diagnostic thresholds and normative reference datasets currently precludes individual-level clinical application.

### 4.6. Limitations

Several limitations of the present synthesis warrant consideration. First, the hybrid design integrates systematic reviews and original cohort-level investigations. While this approach allows both breadth and specificity, it introduces conceptual heterogeneity. Review-level evidence synthesizes diverse populations and analytic frameworks, whereas original studies reflect specific cohorts and preprocessing strategies. Although these components were analyzed separately, complete methodological harmonization is not achievable.

Second, the vote-counting strategy used to summarize network-level convergence does not incorporate effect-size magnitude, statistical power, or confidence intervals. The inferential strength of the present synthesis is therefore inherently limited by the descriptive design, the relatively small number of included studies, and substantial methodological heterogeneity. Consequently, the relative frequency of reported alterations should not be interpreted as the quantitative weighting of biological relevance.

Third, resting-state fMRI relies on blood-oxygen-level-dependent (BOLD) signal fluctuations, which are influenced by neurovascular coupling. Post-traumatic alterations in cerebrovascular reactivity may affect connectivity estimates independently of neuronal synchrony [20]. Without concurrent perfusion-sensitive or vascular reactivity measures, disentangling neuronal from hemodynamic contributions remains challenging.

Fourth, substantial variability in preprocessing pipelines—including motion correction thresholds, global signal regression, filtering parameters, and parcellation schemes—limits direct comparability across studies. Motion-related artifacts, in particular, remain a known confound in functional connectivity research [22]. Potential publication bias toward positive rs-fMRI findings may additionally influence the apparent convergence of reported network alterations.

Fifth, most investigations included were cross-sectional. Longitudinal imaging studies examining network evolution from acute to chronic phases remain relatively limited, constraining inference regarding temporal dynamics of connectivity alterations.

Finally, the qualitative symptom–network mapping was exploratory and descriptive. It does not imply causality or direct predictive validity at the individual patient level. The vote-counting approach does not account for effect-size magnitude, statistical power, or study-level quality weighting, and therefore should be interpreted as a descriptive indicator of convergence rather than a quantitative measure of association strength.

### 4.7. Future Directions

Future investigations should prioritize longitudinal, multimodal designs integrating diffusion imaging, perfusion-sensitive techniques, and task-based paradigms. The standardization of preprocessing pipelines and adoption of open-data frameworks may enhance reproducibility. Integration of connectivity metrics with clinically meaningful outcomes will be essential to determine translational relevance.

## 5. Conclusions

The present hybrid synthesis supports a network-based conceptualization of mild traumatic brain injury. Convergent findings across systematic reviews and original rs-fMRI investigations indicate the preferential involvement of the default mode network, executive frontal circuits, and limbic structures.

Although methodological heterogeneity remains considerable, the recurring pattern of large-scale network disruption suggests that mTBI affects distributed functional integration rather than producing solely focal structural abnormalities. However, current evidence does not yet support direct clinical application of resting-state connectivity metrics for diagnosis or prognostication.

Future longitudinal, multimodal, and standardized imaging frameworks will be essential to clarify the neurobiological mechanisms underlying directional heterogeneity and to determine the translational value of network-level biomarkers in mTBI.

## Figures and Tables

**Table 1:** Systematic reviews and meta-analyses included in the umbrella component.

Study	Year	Modality	Study Type	Scope/Sample	Principal Findings
Aoki et al. [8]	2012	DTI	Meta-analysis	13 DTI studies; 280 mTBI/244 controls	Consistent fractional anisotropy (FA) reductions in corpus callosum; evidence of diffuse axonal injury.
Eierud et al. [5]	2014	DTI, fMRI, sMRI	Systematic review	46 neuroimaging studies (multimodal)	Alterations in default mode and frontoparietal networks across modalities.
Asken et al. [9]	2017	DTI	Systematic review	DTI studies across civilian, military, and sport populations	Marked heterogeneity in white matter findings across mTBI populations.
Dogra et al. [10]	2024	rs-fMRI	Systematic review	Resting-state fMRI studies in mTBI (review-level synthesis)	Inconsistent functional connectivity findings; methodological variability highlighted.

## Data Availability

No new data were created or analyzed in this study.
